# Supplementary material for: AMPA-receptor specific biogenesis complexes control synaptic transmission and intellectual ability
Source: Nat Commun. 2017 Jul 4;8:15910. doi: 10.1038/ncomms15910 (PMC5500892; doi:10.1038/ncomms15910)
Supplement: Supplementary Information [file ncomms15910-s1.pdf]

Type of file: PDF
Size of file: 0 KB
Title of file for HTML: Supplementary Information
Description: Supplementary Figures and Supplementary Tables.

Type of file: XLSX
Size of file: 0 KB
Title of file for HTML: Supplementary Data 1
Description: AMPA receptor molecular abundance values (a.u, normalized to the respective target) in APs underlying the heatmap in Figure 1A.

Type of file: PDF
Size of file: 0 KB
Title of file for HTML: Peer Review File
Description:

Supplementary Information

Supplementary Figure 1 (information related to Fig. 1)

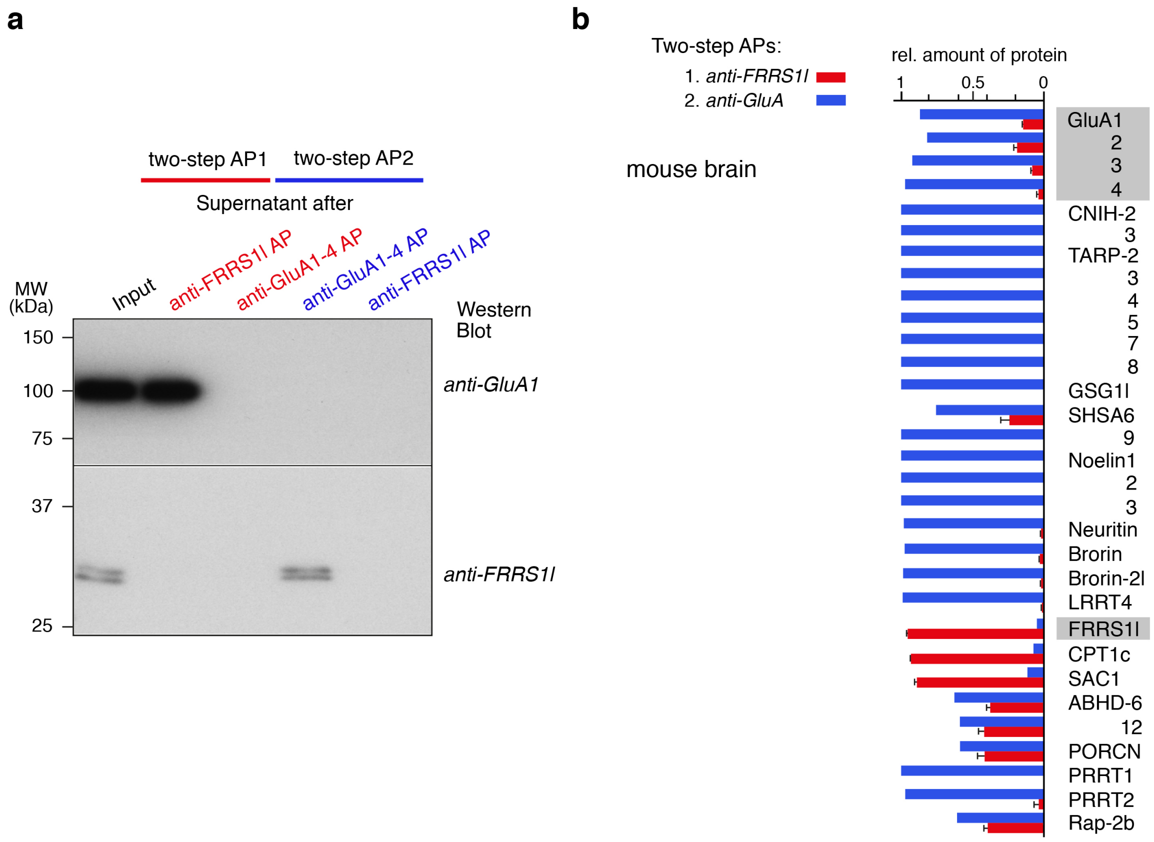

Supplementary Figure 1

Distinct populations of AMPAR assemblies identified by two-step APs in the mouse brain.

(**a**) SDS-PAGE separation and Western blot analysis of the supernatants obtained after depleting APs in the two-step AP experiments (Fig. 1b) demonstrating depletion efficiency.

(**b**) Relative amounts of AMPAR constituents determined in a two-step AP as in Figure 1b but with CL-47 solubilized membrane fractions from mouse brains. Bars depict the relative amounts of AMPAR constituents determined in a target-depleting *anti-FRRS1l* AP (red bars, mean (± SD) of three measurements) that was followed by a target-depleting *anti-GluA* AP (blue bars). For each constituent total amount of protein is given as the summed amounts obtained in the two APs.

Supplementary Figure 2 (information related to Fig. 3)

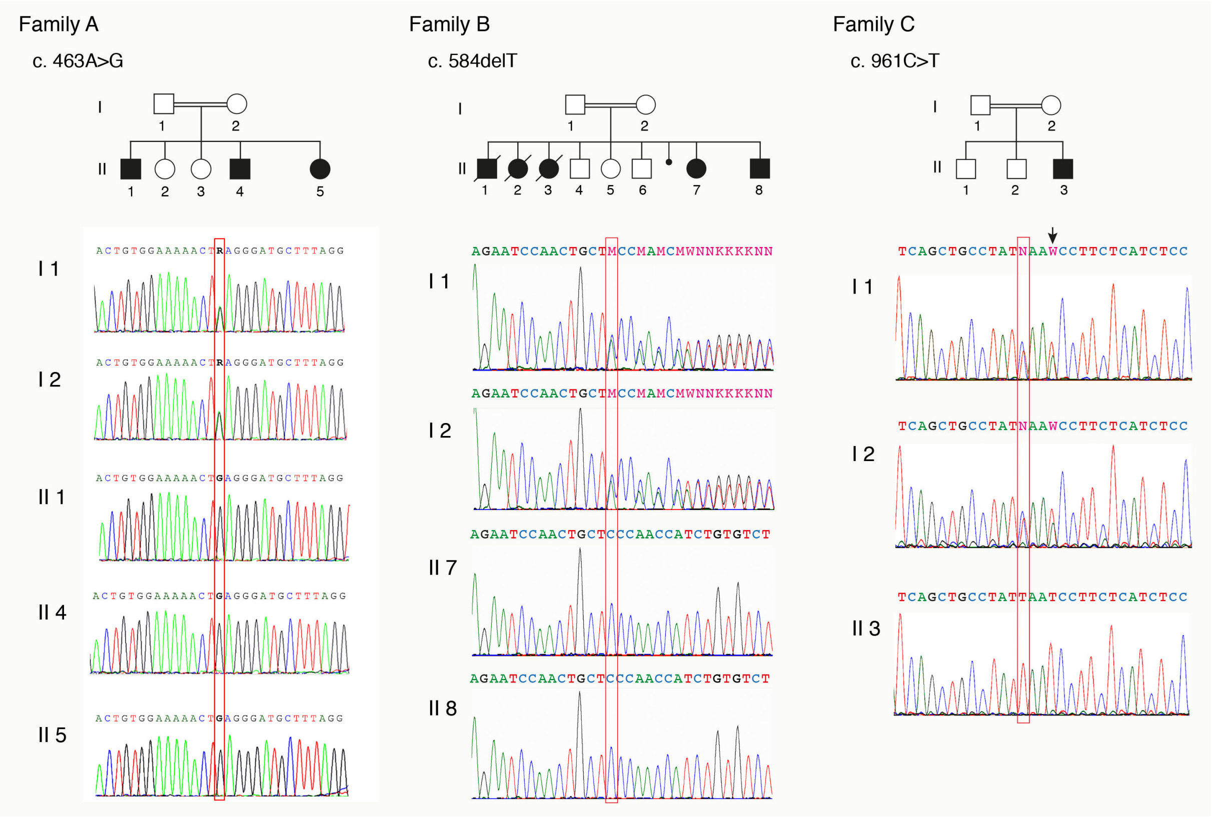

Supplementary Figure 2

Electrophoretograms illustrating the *FRRS1L* variants identified in families A-C.

Data are shown for homozygous affected individuals (lower panels) and heterozygous healthy parents (upper two panels). Red frames indicate the position of the single nucleotide change. For family C an additional missense variant (marked by an arrow head; 9:111899806 A/T; c.964T>A, p. T322S) is detected three nucleotide downstream of the c.961C>T variant (red box). This variant is not described in ExAc; we considered this variant irrelevant since it occurred downstream of the c.961C>T, p.Q321* variant, and the segregation analysis did not suggest a complex rearrangement at this position.

Supplementary Figure 3 (information related to Fig. 4)

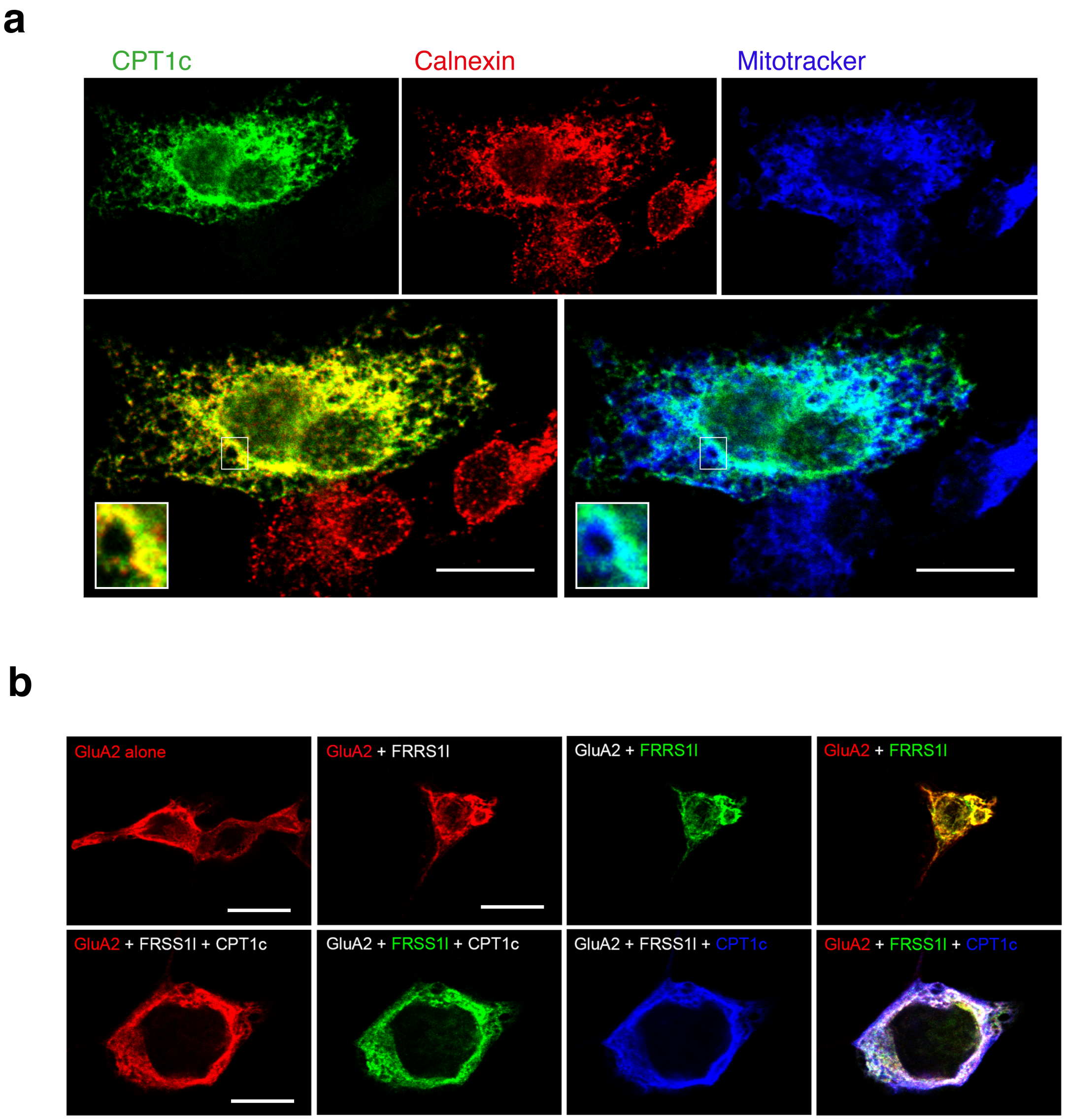

Supplementary Figure 3
ER-localization of CPT1c and co-localization of FRRS1l, GluA2 and CPT1c in the ER.
(**a**) Confocal fluorescence images of tsA-201 cells expressing CPT1c. Upper panel: Fluorescence staining as indicated by the color-coding in the headline. Lower panel: Merged images of CPT1c and calnexin staining (left) and CPT1c and mitotracker (right). Insets: Framed boxes at enlarged scale. Scale bar is 10 µm. Note selective co-localization of CPT1c with the ER marker calnexin, but not with the adjacent mitochondria.
(**b**) Confocal fluorescence images of tsA-201 cells expressing GluA2 (R edited) alone (upper left), together with FRRS1l (upper panel rest) or together with FRRS1l and CPT1c (lower panel). Scale bars are 10 µm. Note close co-localization of both GluA2/FRRS1l and GluA2/FRRS1l/CPT1c.

Supplementary Figure 4 (information related to Fig. 4)

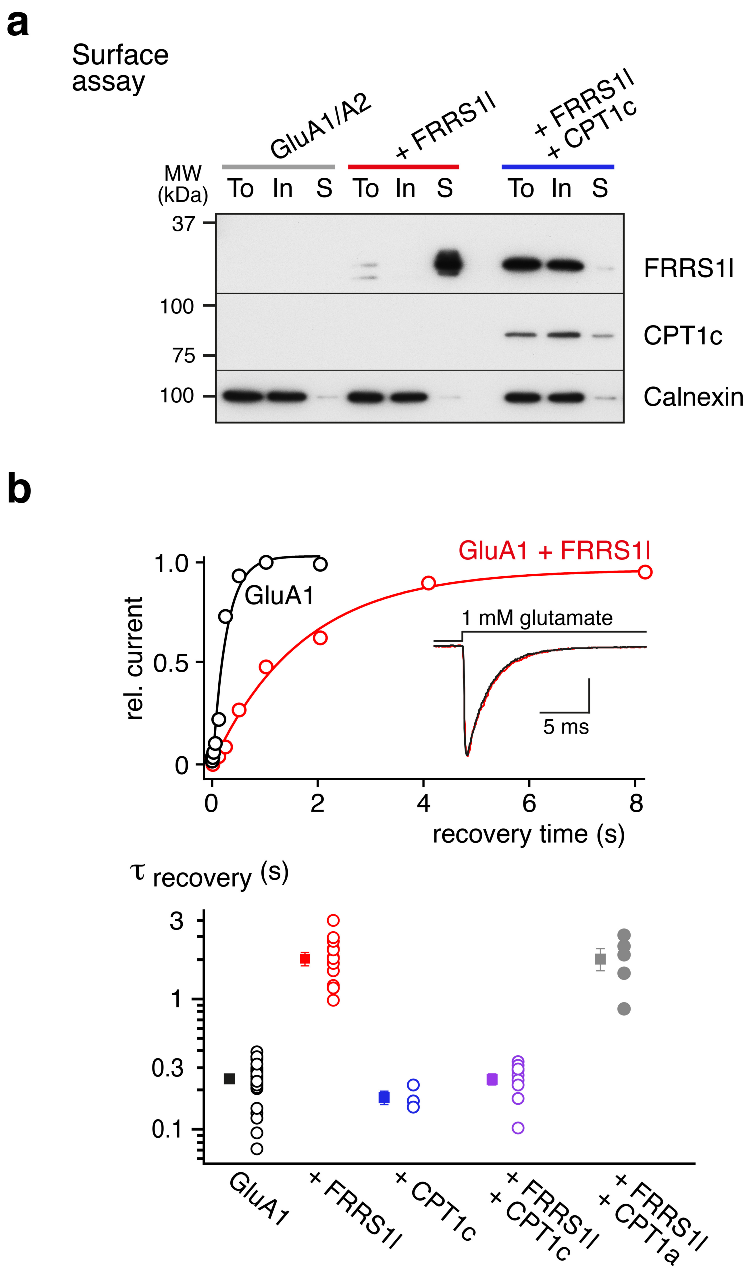

Supplementary Figure 4
Redistribution of FRRS1l by CPT1c.

(**a**) SDS-PAGE separation of distinct pools of membrane fractions that were obtained from surface-biotinylated tsA-201 cells (biotinylation performed in intact cells) expressing the indicated proteins and Western-probed for the proteins indicated on the right. To is total membranes, S is surface membranes (retained by Neutravidin-matrix; load is enriched 12.5-fold), In is intracellular membranes (not retained on Neutravidin-matrix). Note that FRRS1l was only biotinylated in the absence of CPT1c (strong enrichment in S fraction), while in the presence of CPT1c FRRS1l was effectively retained in intracellular membranes (in In fraction, but not in S fraction).

(b) Recovery of GluA1 and GluA1+FRRS1l AMPARs from steady-state desensitization recorded with a double-pulse protocol. Data points are maximal

currents of the test-pulse normalized to the maximal current of the desensitization-inducing pre-pulse; lines are mono-exponential fits with time constants of 264 ms (GluA1) and 1696 ms (GluA1+FRRS1l). Inset: Normalized current responses recorded from GluA1 (black) and GluA1+FRRS1l (red). Lower panel: Recovery time constants and means (± SEM) thereof obtained with AMPARs assembled from the indicated subunits. Note that CPT1c effectively prevents surface delivery of FRRS1l-containing AMPARs, different from CPT1a.

Supplementary Figure 5 (information related to Fig. 4)

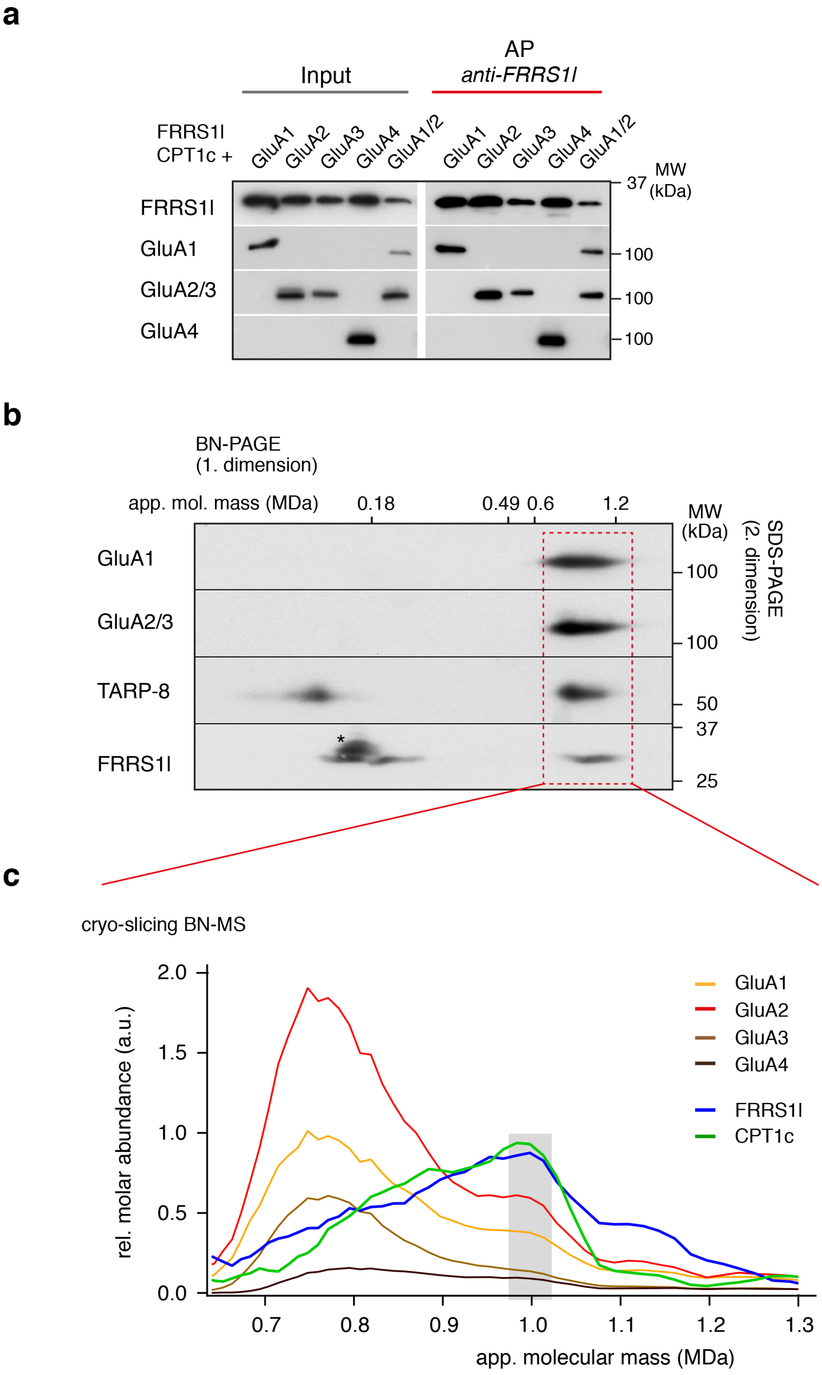

Supplementary Figure 5
Assembly of FRRS1l/CPT1c with GluA tetramers

(**a**) SDS-PAGE separation of input and eluate of an *anti-FRRS1l* AP on membrane fractions prepared from tsA201 cells expressing the indicated proteins Western-probed with ABs targeting FRRS1l, GluA1, GluA2/3 and GluA4. Note robust co-assembly of all GluAs with FRRS1l and CPT1c.

(**b**) Two-dimensional gel separation of membrane fractions prepared from adult rat brain and Western-probed for the indicated proteins. Asterisk denotes target-unrelated binding of the *anti-FRRS1l* AB.

(**c**) Abundance-mass profiles of the indicated proteins obtained from cryo-slicing BN-MS analysis (Müller et al., 2016) of the native gel in the molecular weight range framed in (b); protein abundances were obtained by the QConCAT method and are given as relative molar abundance. Note that FRRS1l and CPT1c display very similar profiles indicating co-assembly with GluAs at an equimolar ratio. Ratios determined for FRRS1l (or CPT1c) and GluAs1-4 in the mass-range shaded in grey (maximum of FRRS1l/CPT1c) yielded values of around 1 (1.05 – 1.25), in line with roughly four FRRS1l/CPT1c complexes being co-assembled with each GluA tetramer.

Supplementary Figure 6 (information related to Fig. 4)

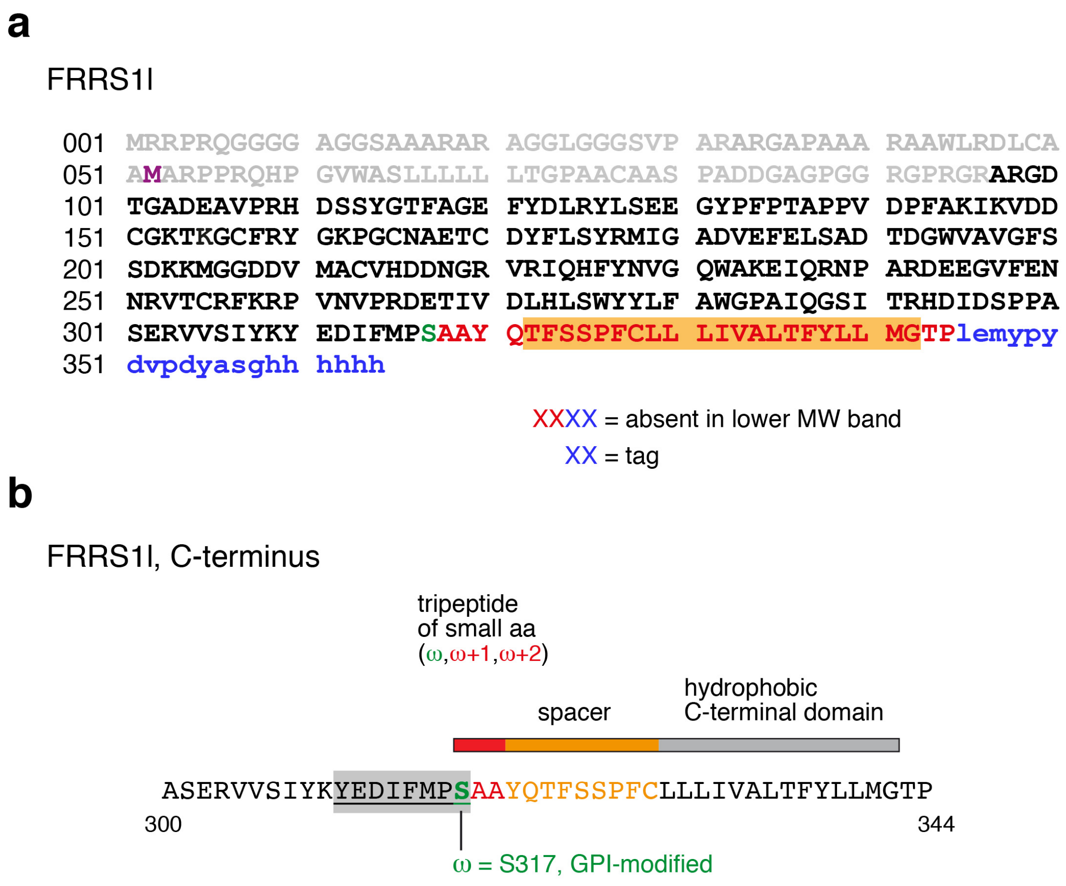

Supplementary Figure 6

Analysis of primary sequence and C-terminal modification of FRRS1l.

(**a**) Coverage of the primary sequence of FRRS1l (human) as obtained in CID MS/MS-analysis following digestion with trypsin and alpha-lytic proteases (WaLP and MaLP, see Experimental Procedures). Sequences identified by mass spectrometry are in black, green, red and blue, those not identified are in grey. Orange box denotes the hydrophobic domain, the epitope-tags are indicated in blue. S317 is highlighted in green, the methionine most likely used as a translation start is indicated in purple. Note that the amino acids in red and blue were only detected in the non-cleaved form of FRRS1l (higher MW band in Figure 4C). (**b**), Scheme illustrating the C-terminal sequence motifs related to GPI-modification as given in (Mayour and Riezma, 2004; Galian et al., 2012).

Supplementary Figure 7 (information related to Fig. 6)

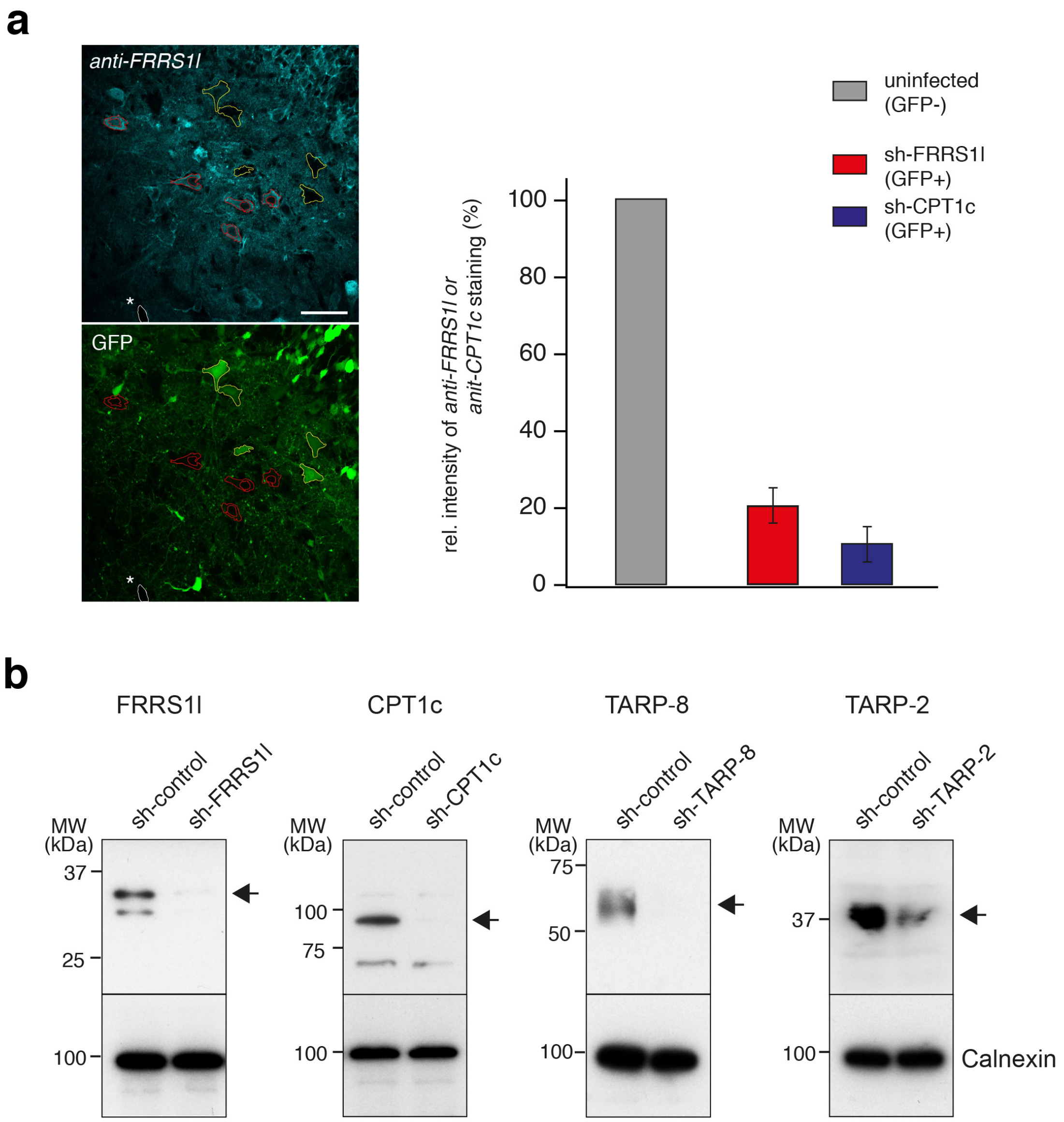

Supplementary Figure 7
Properties of sh-RNAs used in this study.
(**a**) Efficacy and specificity of sh-FRRS1l and sh-CPT1c lentivirus in slice preparations obtained from stereotactically injected rats (Boudkkazi et al., 2014). Left panel: Confocal fluorescence images of a hilar hippocampal slice section. Fluorescence shown in cyan (upper image) originates from staining with an *anti-FRRS1l* antibody *(visualized* with an Alexa633-conjugated secondary antibody), GFP-fluorescence (lower image) indicates cells transduced with the sh-FRRS1l virus. Areas framed with lines denote FRRS1l-fluorescence (red line) or GFP-fluorescence (yellow line) in soma and proximal dendrites; the lines were manually drawn in the proper z-stack on the respective fluorescence channel and projected onto both fluorescence images. Right panel: Bar graph summarizing mean ± SEM of the *anti-FRRS1l* and the *anti-CPT1c* staining intensity determined in 7 and 6

cells from 3 randomly selected slices from different rats; staining intensity of each transduced cell (GFP-positive) was normalized to the mean value obtained from 6-11 untransduced control cells expressing FRRS1l or CPT1c in each slice preparation (GFP-negative). All intensity values were corrected for background fluorescence (frame in white marked with an asterisk). (**b**) Membrane fractions of tsA-201 cells expressing the indicated target protein in the presence of sh-control or the indicated target sh-RNA separated by SDS-PAGE and Western probed with antibodies specifically recognizing the respective target protein (marked with arrowhead).

Supplementary Figure 8 (information related to Figs. 6, 7)

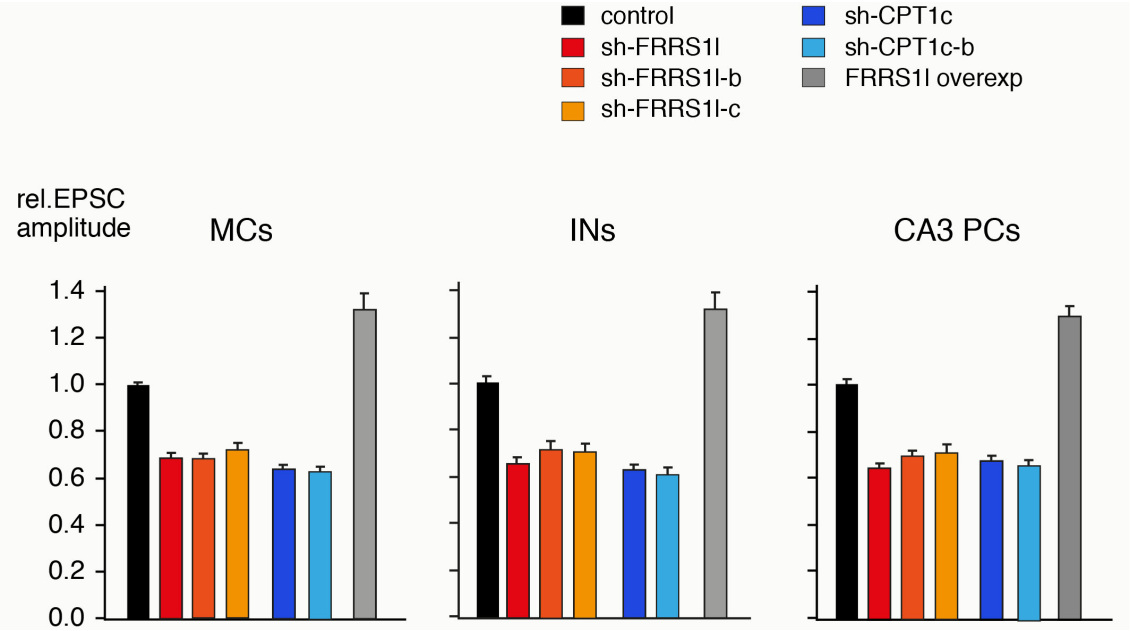

Supplementary Figure 8
Effect of protein knockdown and (over) expression on EPSC amplitudes.
Relative amplitudes of spontaneous EPSCs measured in the three types of hilar neurons as in Figure 6 with the indicated sh-RNAs targeting FRRS1l and CPT1c. Data for sh-FRRS1l-b, -c and sh-CPT1c-b are mean ± SEM from 11-32 MCs, 17-54 INs and 27-51 CA3 PCs. Data for control, sh-FRRS1l, sh-CPT1c and over-expression of FRRS1l (from Figure 6B) were added for better comparison.

Supplementary Figure 9 (information related to Fig. 7)

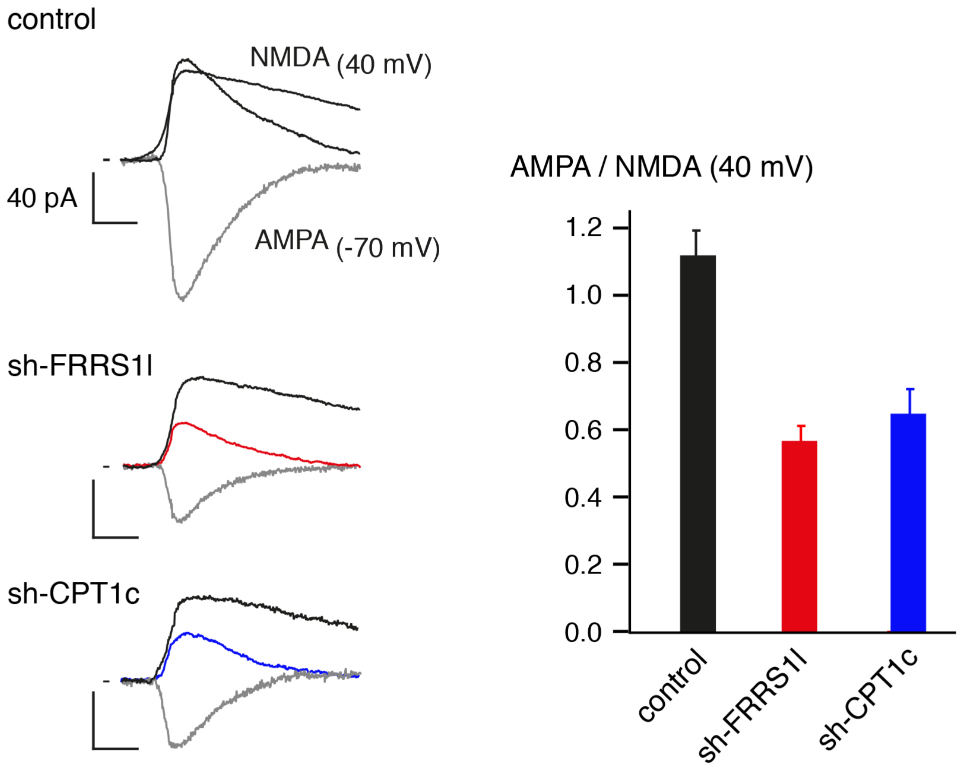

Supplementary Figure 9

Decrease in AMPA/NMDA ratio mediated by sh-FRRS1l and sh-CPT1c.

Left panel: Spontaneous EPSCs through NMDARs at a membrane potential of 40 mV (with AMPAR-mediated components of the EPSCs blocked by 20 µm CNQX) and through AMPARs at membrane potentials of 40 mV (distinguished by the kinetics) and -70 mV (grey) recorded in uninfected MCs (upper part) or MCs transduced with sh-FRRS1l or sh-CPT1c (middle and lower parts). Time scaling is 10 ms. Right panel: Bar diagram summarizing amplitude ratios of NMDAR- and AMPAR-mediated EPSCs (at 40 mV) obtained from experiments as on the left panel. Data are mean ± SD of 9-10 MCs.

Supplementary Figure 10

Figure 4a

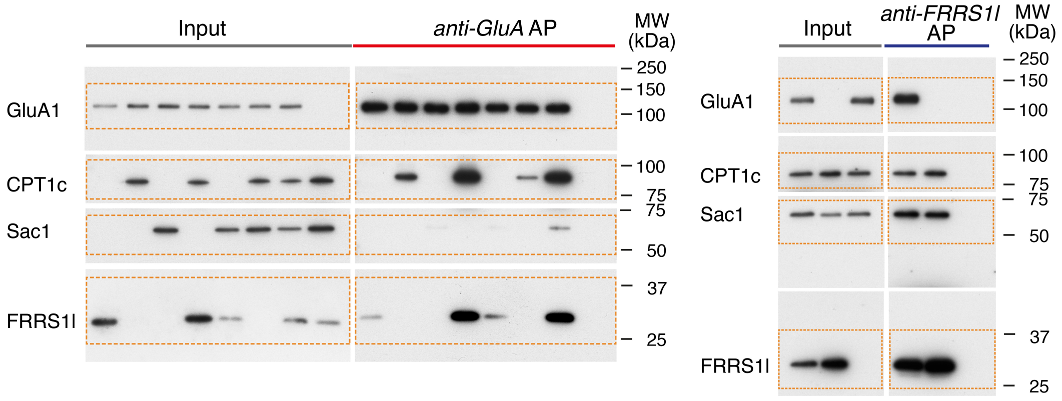

Figure 4c

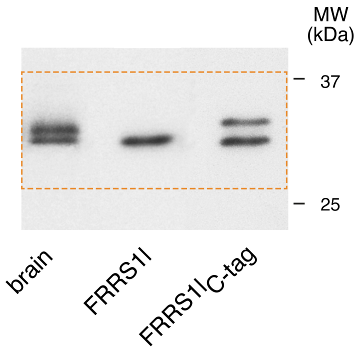

Figure 4d

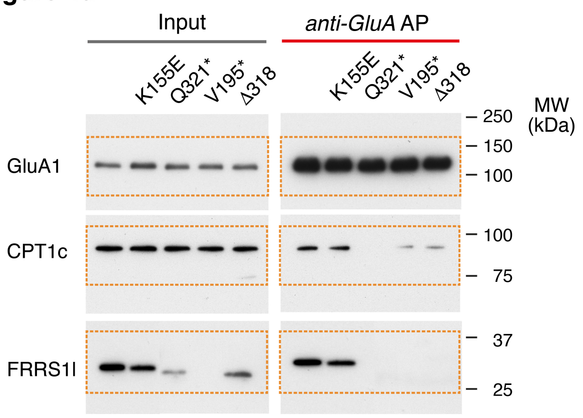

Supplementary Figure 1

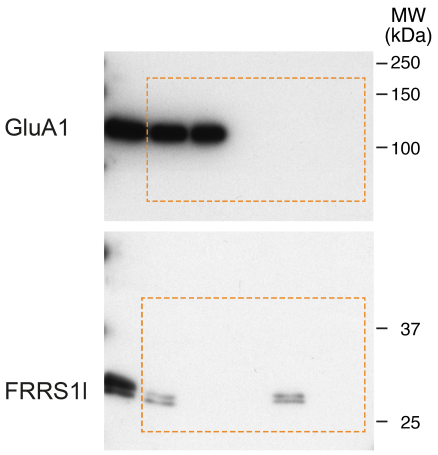

Supplementary Figure 4a

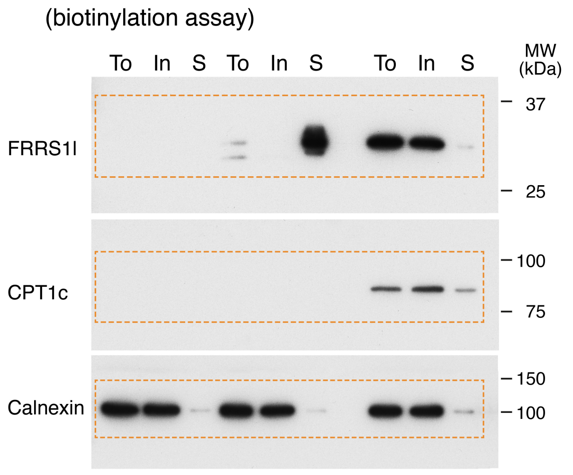

Supplementary Figure 10
Western blot images in full size.
Western blot data shown in Fig. 4 and Supplementary Figures 1 and 4a at full size. Sections boxed by yellow dashed lines approximate the region illustrated in the aforementioned figures.

Supplementary Figure 11

Supplementary Figure 5

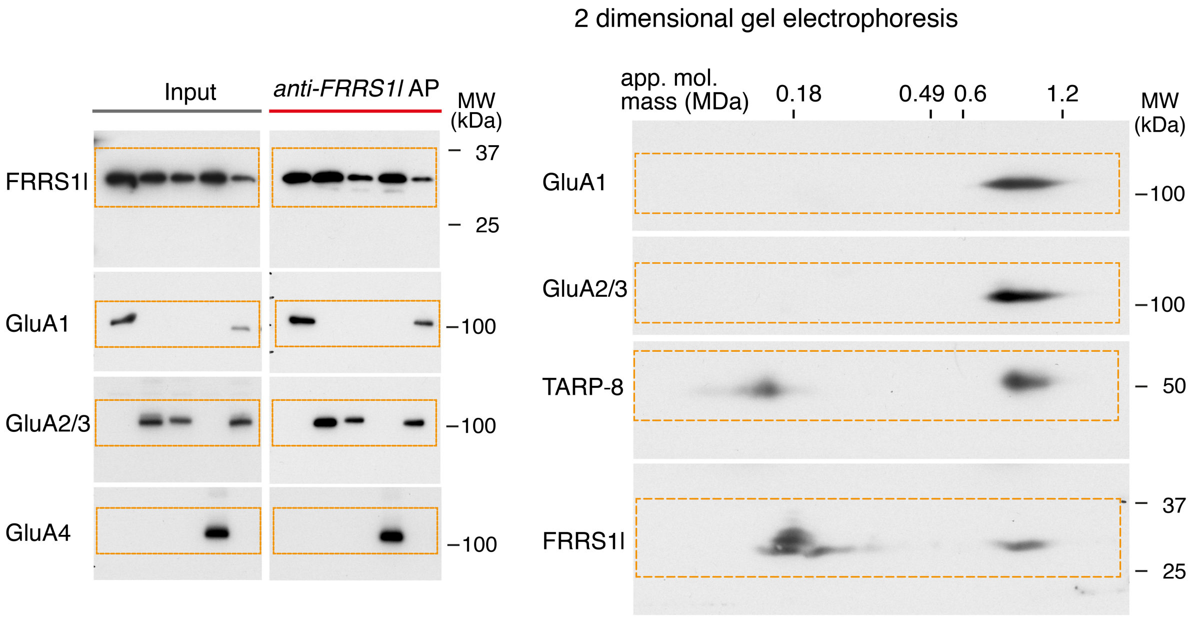

Supplementary Figure 8

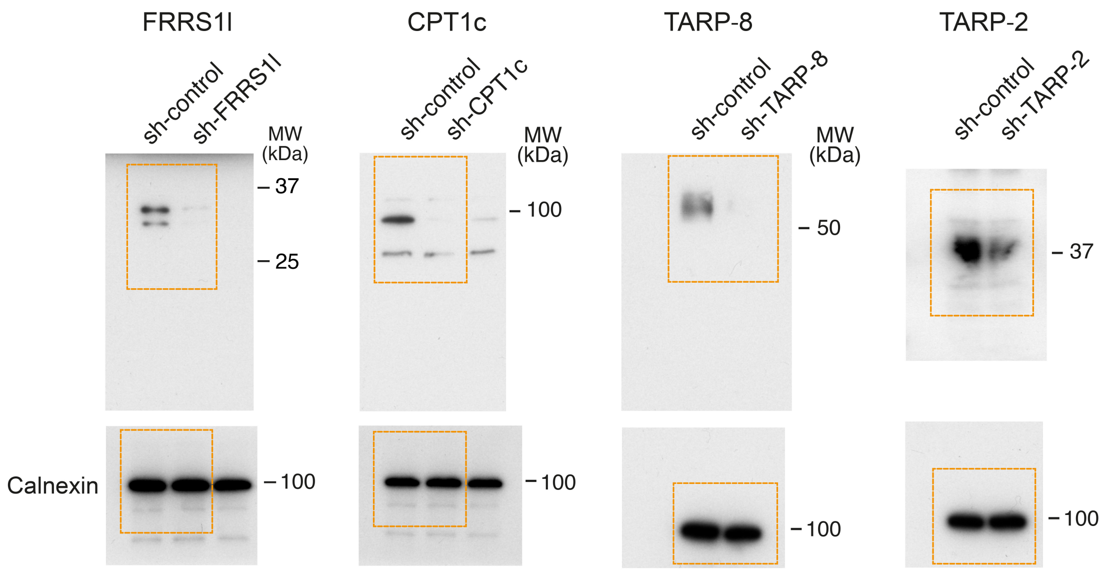

Supplementary Figure 11
Western blot images in full size.
Western blot data shown in Supplementary Figures 5 and 8 at full size. Sections boxed by yellow dashed lines approximate the region illustrated in the aforementioned figures.

Supplementary Table 1 (information related to Fig. 3)

Suppl. Table 1: Summary of WES and filtering processes

Family A			
Patients	II.1	II.4	II.5
Sequencing platform		HiSeq 2500	
Total variants	94454	96916	96664
Novel or rare variants (dbSNP130/1000GP/EVS/in-house database)	504	565	544
Coding Non-synonymous/indel/consensus SS variants	126	142	136
Shared variants		47	
Shared variant segregating in the family		1	

Family B	
Patients	II.8
Sequencing platform	SOLID 4
Total variants	51756
Novel or rare variants (dbSNP130/1000GP/EVS/in-house database)	10642
In candidate regions	37
Coding Non-synonymous/indel/consensus SS variants	2

Family C			
Patient / parents	II.3	I.1	I.2
Sequencing platform		Proton	
Total variants	61232	64456	62348
Novel or rare variants (dbSNP130/1000GP/EVS/in-house database)	14588	14276	13861
Homozygous in index and heterozygous in parents		87	
Coding Non-synonymous/indel/consensus SS variants		27	
MAF < 2% in house probes		3	
In genes relevant to the phenotype		1	

Supplementary Table 1
Number of variants identified by WES and overview of the filtering processes used. For Family B, WES identified two rare variants fulfilling the filtering criteria: *FKTN* (MIM 607440; NM_006731:c.706A>G; p.M236V) and *FRRS1L* (MIM 604574; NM_014334:c.584delT; p.V195E fs*35). Mutations in *FKTN* cause muscular dystrophy-dystroglycanopathy (congenital with brain and eye anomalies), type A4 (MIM 253800). Affected individuals present with muscular dystrophy, heart defects, eye abnormalities, brain malformations and intellectual disability. This variant is

poorly conserved and predicted benign by in-silico prediction programs. Moreover, neither heart defects nor eye abnormalities were observed in affected individuals of family B. Thus, we classified this variant as likely non-causative for the phenotype of the affected individuals.

For family C, the three variants remaining after filtering were: *FRRS1L*: NM_014334.2: c. 961C>T: p. Q321*), *FRRS1L*: NM_014334.2: c. 964A>T: p. T322S), and *DDIT4L*: NM_145244.3: c. 136G>A: p. E46K). While we considered the missense variant in *FRRS1L* irrelevant as it occurs downstream of the nonsense mutation, the variant in *DDIT4L* was evaluated in detail. This variant, rs374110796 is reported four times in the ExAc database, and once in the ESP database. Several publications report abundant expression of this gene in skeletal muscle and suggest participation in the regulation of mTOR signaling (e.g. Miyazaki and Esser, 2009; Kelleher et al., 2014). As yet, there is no associated phenotype described. Although a potential contribution of the *DDIT4L* variant to the observed phenotype cannot be entirely excluded, we considered *FRRS1L* as the more relevant candidate gene based on both genetic (minor allele frequency is 0 and a nonsense mutation) and clinical data (homozygous variants in *FRRS1L* segregating in 5 other patients from 2 families with an overlapping phenotype).

Supplementary Table 2: Cinical characteristics of patiens with FRRS1l mutations

family	A			B		C
patient	II-1	II-4	II-5	II-7	II-8	II-3
**mutation**	c.463A>G p.K155E	c.463A>G p.K155E	c.463A>G p.K155E	c.584delT p.V195E fs*35	c.584delT p.V195E fs*35	c.961C>T p.Q321*
gender	m	m	f	m	m	m
age (yrs.) of examination	21	14	13	3	1.5	2
**intellectual disability**	++ (MA 8 yrs.) WAIS-III	++ (MA 6 yrs.) WAIS-III	+++ (MA 7 yrs.) WISC-IV	+++	+++	+++
**motor skills**						
sit	NA	NA	NA	not achieved	not achieved	not achieved
walk	24 months	16 months	12 months	not achieved	not achieved	not achieved
**speech development**	delayed	delayed	delayed	single words	single words	not achieved
**neuro-regression**	–	–	–	+	+	+
**epilepsy** (age of onset)	+ (6 yrs.)	+ (13 yrs.)	+ (5 yrs.)	+	+	+
**CT scan** (brain)	normal	normal	normal	NA	NA	normal
**MRI** (brain)	normal	NA	normal	NA	NA	normal
**muscular hypotonia**	–	–	–	+	+	+
**growth parameters**						
head circumference	54 cm (-1.5 SD)	51 cm (-2 SD)	50 cm (-3 SD)	47 cm (-1.6 SD)	48.5 cm (0.8 SD)	50 cm (1.3 SD)
body height	154 cm (-3 SD)	142 cm (-2 SD)	131 cm (-3 SD)	94 cm (3 SD)	91 cm (3 SD)	97 cm (-0.5 SD)
body weight	48 kg	36 kg	35 kg	12.9 kg	14 kg	13.6 kg

WAIS: Wechsler Adult Intelligence Scale, WISC: Wechsler Intelligence Scale f. Children; MA: Mental Age
